# Protein Cage Directed Assembly of Binary Nanoparticle Superlattices

**DOI:** 10.1002/advs.202408416

**Published:** 2024-10-14

**Authors:** Yu Zhou, Ahmed Shaukat, Jani Seitsonen, Carlo Rigoni, Jaakko V. I. Timonen, Mauri A. Kostiainen

**Affiliations:** ^1^ Biohybrid Materials Department of Bioproducts and Biosystems Aalto University Aalto 00076 Finland; ^2^ School of Biological and Biomedical Sciences Durham University Durham DH13LE UK; ^3^ Nanomicroscopy Center Aalto University Aalto 00076 Finland; ^4^ Department of Applied Physics Aalto University School of Science Aalto University Aalto 00076 Finland; ^5^ Institute of Science and Technology Austria Am Campus 1 Klosterneuburg 3400 Austria

**Keywords:** binary crystal, hierarchical self‐assembly, metal nanoparticle, protein cage, superlattice

## Abstract

Inorganic nanoparticles can be assembled into superlattices with unique optical and magnetic properties arising from collective behavior. Protein cages can be utilized to guide this assembly by encapsulating nanoparticles and promoting their assembly into ordered structures. However, creating ordered multi‐component structures with different protein cage types and sizes remains a challenge. Here, the co‐crystallization of two different protein cages (cowpea chlorotic mottle virus and ferritin) characterized by opposing surface charges and unequal diameter is shown. Precise tuning of the electrostatic attraction between the cages enabled the preparation of binary crystals with dimensions up to several tens of micrometers. Additionally, binary metal nanoparticle superlattices are achieved by loading gold and iron oxide nanoparticles inside the cavities of the protein cages. The resulting structure adopts an AB_2_^FCC^ configuration that also impacts the dipolar coupling between the particles and hence the optical properties of the crystals, providing key insight for the future preparation of plasmonic and magnetic nanoparticle metamaterials.

## Graphical abstract

Co‐crystallization of two different protein cages characterized by opposing surface charges and unequal diameter is shown. Encapsulating metal nanoparticles inside the cages and precise tuning of the electrostatic attraction between the cages enables the preparation of binary crystals with dimensions up to several tens of micrometers.

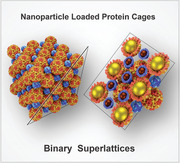

## 1 Introduction

Combining material science, nanotechnology, and biological systems offers a trajectory to unlock next‐generation bio‐inspired materials and molecular devices.^[^ ^1^ ^]^ Their development relies on the intricate art of programming nanoscale structural order into complex matter, and the successful combination of multi‐functional synthetic materials with highly uniform biomacromolecules.^[^ ^2^, ^3^ ^]^

Protein cages are sophisticated biomolecular assemblies with well‐defined structures and hollow cavity, which can be used for compartmentalization of biological functions.^[^ ^4^ ^]^ Protein cages can be repurposed for a wide range of nanomaterials, including drug delivery vehicles,^[^ ^5^, ^6^, ^7^, ^8^ ^]^ imaging agents,^[^ ^9^, ^10^ ^]^ nanoreactors,^[^ ^11^, ^12^, ^13^, ^14^, ^15^ ^]^ vaccines^[^ ^16^, ^17^, ^18^ ^]^ and diverse nanoparticle arrays.^[^ ^19^, ^20^, ^21^, ^22^ ^]^ For instance, protein cages have demonstrated their versatility by forming binary crystals in biocompatible environments with gold nanoparticles,^[^ ^20^, ^23^ ^]^ small proteins,^[^ ^24^, ^25^ ^]^ dendrimers,^[^ ^26^, ^27^, ^28^ ^]^ and synthetic molecules.^[^ ^29^, ^30^ ^]^ However, there is limited research on the formation of binary crystals composed exclusively of protein cages. Such an approach avoids the dispersity related to synthetic components and can increase the quality of the resulting crystals. Only a few such studies exist, where protein cage based binary lattices consisting of for example genetically engineered supercharged ferritins are crystallized with other cages.^[^ ^19^, ^31^, ^32^, ^33^ ^]^ Despite these advances, a significant gap persists in exploring nanoparticle superlattices formed using differently sized protein cages, particularly in achieving ordered superlattice structures with synergistic coupling interactions between various nanoparticles inside them.

**Figure 1:**
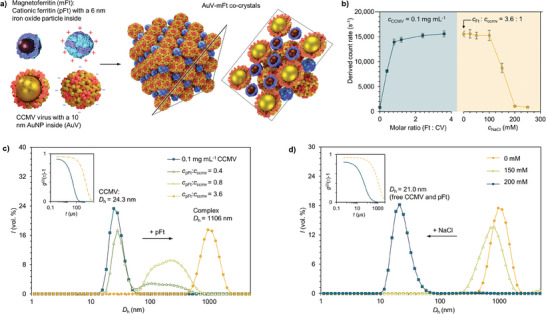
Self‐assembly of CCMV–pFt complexes at pH 4.8. a) Schematic representation of the formation of heterogeneous binary crystals, composed of AuV and mFt. b) Left: light scattering intensity of a CCMV solution titrated with an increasing concentration of pFt indication complex formation. Right: The complexes were disassembled by increasing the ionic strength of the medium by the addition of NaCl. c) Dynamic light scattering (DLS) data for the volume‐averaged size distribution of CCMV titrated with an increasing amount of pFt and d) the resulting complexes disassembled with NaCl. Insets: second‐order autocorrelation functions of the corresponding measurements.

Here, we show that protein cages possessing oppositely charged surface charges and different sizes – specifically, negatively charged wild type cowpea chlorotic mottle virus (CCMV) and an engineered positively charged ferritin (pFt) – serve as effective building blocks for the self‐assembly of intricate 3D binary superlattices (**Figure** **1**a). The negatively charged CCMV is loaded with gold nanoparticles (AuV) and co‐crystallized with pFt cages, which accommodate iron oxide nanoparticles (mFt). The assembly kinetics can be controlled by varying the electrolyte concentration and hence the range of electrostatic interactions. Varying the assembly conditions induces subtle changes on the assembly process, consequently giving rise to the formation of distinct crystal structures. For example, the co‐assembly of native CCMV viruses and empty pFt cages yields an AB_6_^BCC^ type crystal structure. Intriguingly, regardless of which protein cage is loaded with nanoparticles, an AB_2_^FCC^ type crystal structure emerges. The obtained protein cage directed gold nanoparticle – iron oxide nanoparticle co‐crystals are relatively large, with domain sizes up to 20 µm. This avenue of investigation holds the promise of untangling the intricate interplay between structural arrangement and the manifestation of distinct biomaterial properties.

## 2 Results and Discussion

CCMV is a compact plant virus measuring ≈28 nm in outer diameter and 18 nm in inner diameter. The capsid comprises of 180 identical coat protein subunits, which adopt a Caspar–Klug T = 3 quasi‐icosahedral symmetry and enclose a positive‐sense single‐stranded RNA (+ssRNA) segmented genome of ≈ 3 000 nucleotides.^[^ ^34^ ^]^ Our second building block is human heavy chain ferritin, well‐known for its native function in iron mineralization and storage.^[^ ^35^ ^]^ It is constructed from 24 protein subunits, forming a hollow spherical shell with octahedral symmetry and an outer diameter of 12 nm and an inner diameter of 8 nm.^[^ ^36^ ^]^Considering the negative outer charge of both of the native protein cages, we engineered a positively charged ferritin variant through an established surface modification approach to facilitate the electrostatic co‐crystallization with CCMV at neutral pH (Figure 1a). Nine lysine or arginine amino acid residues were added per protein subunit to yield a cationic ferritin mutant termed (pFt), with a notably positive exterior surface.^[^ ^37^ ^]^

Initially, dynamic light scattering (DLS) was used to investigate the complex formation. This was achieved by monitoring changes in scattering intensity (derived count rate) and hydrodynamic diameter (*D*_h_). The procedure involved titrating pFt into an acetate buffer (20 mm sodium acetate, pH 4.8) containing CCMV (0.1 mg mL^−1^). Upon the addition of pFt, a rapid increase in count rate was observed, indicating the formation of large complexes. The count rate reached its maximum when the protein cages molar ratio was ≈1:1. Further additions of pFt resulted in minimal changes in count rate. To establish the role of electrostatic interactions, NaCl addition was used to disassemble the complexes. A clear decrease in the scattering intensity was observed at concentrations above 200 mm NaCl (Figure 1b). Subsequently, the variations in *D*_h_ provided further evidence of reversible assembly. As pFt concentration increased, the *D*_h_ presented an increase, leading to a reduction in the peak ≈24 nm, corresponding to the original size of CCMV particles. Moreover, at higher pFt concentrations, the diameter of the complexes was measured to be ≈1 000 nm (Figure 1c). Similarly, the addition of 200 mm NaCl led to a restoration of the initial Dh, indicating that an increase in electrolyte concentration effectively disassembled the system back to its original state (Figure 1d).

To investigate if the assembly of CCMV‐pFT complexes is affected by changes in pH, the aforementioned DLS experiments were repeated in neutral pH 7.4 (Figure S2a,b). Titration data shows that at higher pH a higher excess of pFT is required to form large complexes (*D*_h_ > 1 000 nm), which are formed upon the addition of 7.2‐fold molar excess of pFt. This effect arises probably due to CCMV having a higher negative charge under neutral conditions, compared to pH 4.8, while pFt exhibits a lower positive charge at neutral pH (Figure S3). Consequently, CCMV binds with pFt in a wide pH window, but more pFt cages are needed to balance the electrostatic attraction with one CCMV molecule at neutral pH. The complexes formed at pH 7.4 also require a high NaCl concentration (400 mm) for subsequent partial disassembly (Figure S2c), as shown by the count rate data (Figure S2a). This situation can arise due to an overcrowding effect of pFt particles around CCMV, leading to the formation of irreversible complexes.^[^ ^38^ ^]^ To achieve reversible assembly of the two protein cages under neutral pH conditions, we adjusted the order of component introduction in the mixed system and adapted the new strategy for the following measurements. As depicted in Figure S4, we initially prepared a 0.1 mg mL^−1^ pFt solution dispersed in a neutral pH buffer with a predetermined NaCl concentration. In this manner, the presence of NaCl in solution prior to mixing the two protein cages may slow down the overall assembly process, thereby preventing the formation of kinetically trapped structures. Subsequently, CCMV was added to achieve a final concentration of 0.1 mg mL^−1^. The assembled complexes (*D*_h_ > 1 000 nm) can be observed from the solution containing 150 mm NaCl, whereas only free CCMV and pFt particles were present under 250 mm NaCl condition. Taken together the DLS data indicates that the formation of higher‐order structures is predominantly governed by electrostatic interactions and can be controlled by ionic strength as well as pH.

**Figure 2:**
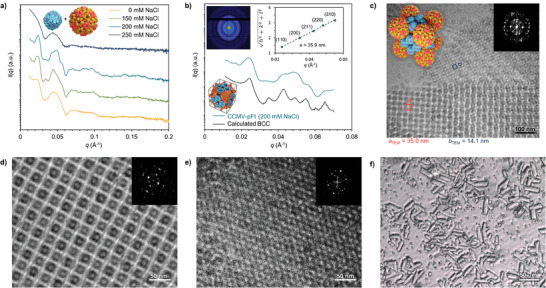
Structural characterization of CCMV–pFt complexes. a) SAXS data was measured for the CCMV–pFt complex at various NaCl concentrations. b) SAXS at 200 mm of NaCl, compared to a computed BCC model (offset along y‐axis for clarity). Inset: 2D scattering pattern (top left) and square root of the sum of the square of the Miller indexes of the assigned reflections for the BCC structure versus the measured q‐vector positions (top right), dashed line presents a linear fit, which yields a lattice parameter *a*_SAXS_ = 35.9 nm (for cubic structures *a *= 2π √(h^2^ + k^2^ + l^2^)/*q*_(hkl)_). c) Cryo‐TEM image of vitrified aqueous solutions containing CCMV–pFt complexes at 200 mm NaCl. Pattern features with measured dimension are outlined with red and blue. The inset shows a model of the AB_6_^BCC^ structure. d,e) High‐magnification cryo‐TEM images of the CCMV‐pFt complexes. Insets: Corresponding fast Fourier transform (FFT). f) Optical microscopy image of tabular CCMV–pFt crystals showing lengths over 30 µm.

Next, we employed small‐angle X‐ray scattering (SAXS) to elucidate the structural morphology of the complexes at different electrolyte concentrations, which has been shown to play a key role in controlling the crystallinity of protein cage assemblies.^[^ ^20^ ^]^ At low NaCl concentration, the complexes have an amorphous morphology as indicated by the lack of well‐defined scattering pattern. Also elevating the electrolyte concentration above 250 mm NaCl shows only the form factor of free cages indicating the presence of free particles, which aligns well with the DLS data. However, the SAXS patterns obtained in the presence of 150–200 mm NaCl exhibit clear Bragg reflections (**Figure** **2**a). The 2D diffraction patterns showed multiple Debye rings, typical of polycrystalline samples, revealing an isotropic alignment of crystals. The azimuthally integrated scattering curves show multiple well‐defined diffraction peaks, indicating relatively long‐range order. SAXS patterns obtained at 150 and 200 mm NaCl are almost similar although the latter shows slightly better translational order. At 200 mm NaCl, the main diffraction peaks are observed at a magnitude of the scattering vector *q* = 0.239, 0.343, 0.425, 0.481, and 0.551 nm^−1^. They were assigned to reflections from crystal planes with Miller indices (hkl) = (110), (200), (211), (220), and (310), which correspond to *q* ^n^/q* = √2, √4, √6, √8, and √10, respectively. The square root of the sum of the squared Miller indexes were plotted against measured *q*(*hkl*) values and fitted with linear regression to yield a lattice parameter of *a* = 35.9 nm (Figure 2b). Based on the SAXS profiles and positions, widths, and relative intensities of the diffraction maxima, coupled with comparison to simulated scattering curves of a finite body‐centered cubic (BCC) structure, CCMV and pFt cages adopt a BCC Bravais lattice (space group *Im3m*, number 229).

Cryogenic‐transmission electron microscopy (Cryo‐TEM) characterization was used to further characterize the nanostructure (Figure 2c; Figure S5a,b). Cryo‐TEM image in Figure 2c shows 3D crystallites in two different orientations. The center‐to‐center distances of the cages for the two patterns were measured as *a*_TEM_ = 35.0 nm and *b*_TEM_ = 14.1 nm, respectively. Subsequently, high‐magnification images along the same projection axes were obtained. CCMV particles are clearly visible in the “a” pattern showing the anticipated cubic arrangement along the [100] projection axis (Figure 2d). The “b” lattice consists of pFt adjacent to the “a” lattice and exhibited a densely packed, approximately hexagonal pattern (Figure 2e). The packing of the cages was further visualized with cryo‐electron tomography (Cryo‐ET). The generated density maps viewed along different projection axes (Figure S5c,d), show the binary structure with particles of two distinct sizes and cubic arrangement. Overall, the observed patterns and dimensions strongly support the presence of a BCC crystal structure.

According to SAXS results, the final BCC structure formed by CCMV‐pFt has a unit cell size of 35.9 nm. Since the SAXS results primarily from CCMV containing the RNA genome, CCMV occupies the lattice points in this unit cell, resulting in a minimum center‐to‐center distance between CCMV particles of *a* √3/2 = 31.1 nm, leaving a 3.1 nm gap between adjacent CCMV particles. The bcc structure includes both tetrahedral and octahedral voids when CCMV is located in the lattice points of the unit cell. The tetrahedral voids in this unit cell have a diameter of 11.5 nm and the octahedral voids have a diameter of 7.2 nm, indicating that pFt can only occupy tetrahedral voids within the unit cell. With this assumption, the minimum distance between adjacent pFt particles within the unit cell is *a*  √2/4 = 12.7 nm, which precisely matches the diameter of pFt particles. We therefore believe that pFt is located at the center of the tetrahedral voids within the BCC unit cell, while CCMV are distributed around the vertex positions yielding an overall AB_6_^BCC^ structure. Inverse Fourier transform calculated with selected Fourier components of the CCMV and pFt layer lattices closely resembled the structural model viewed along the [100] and [110] projection axis separately, largely confirming our assumption (Figure S6). Such crystallographic arrangements have been previously reported, for example, in binary crystals composed of ligand stabilized gold and Fe_3_O_4_ nanoparticles.^[^ ^39^ ^]^ It is noteworthy that large prismatic CCMV‐pFt crystal, up to 30 µm in length could be prepared using the hanging drop crystallization method under 200 mm NaCl condition in three days (Figure 2f).

Protein cages have a hollow interior, which can be used to encapsulate,^[^ ^40^, ^41^ ^]^ protect,^[^ ^42^ ^]^ and deliver various materials of interest.^[^ ^43^, ^44^ ^]^ In our case, the heterologous protein cages of varying sizes can serve as templates for binary crystals with different particle types and dimensions, offering a significant expansion for the available building blocks. We therefore selected gold nanoparticles and iron oxide nanoparticles as model cargo to investigate the preparation of binary superlattices. First, CCMV capsid was utilized to encapsulate DNA‐stabilized 10 nm gold nanoparticles (AuNPs). A 19 nucleotide long single‐stranded (ss) DNA oligo was attached on the AuNPs surface through thiol chemistry using a pH‐assisted and surfactant‐free method.^[^ ^45^ ^]^ The hydrodynamic size of unmodified AuNPs measured by DLS was 13.5 nm, increasing to 15.7 nm with attached DNA (Figure S7a). TEM imaging shows nanoparticles with a uniform size distribution and an average diameter of 14.2 nm, consistent with DLS measurements. However, a small fraction of larger AuNPs (≈18 nm) might impact encapsulation within CCMV cages, potentially leaving them unencapsulated and affecting the subsequent self‐assembly of the binary structure. (Figure S7b,c). DNA coating promotes the interaction with the positively charged N‐terminus of CCMV capsid proteins enabling encapsulation.^[^ ^46^, ^47^ ^]^ Figure S8a shows that the hydrodynamic diameter of the synthesized AuNPs loaded CCMV (AuV) particles (*D*_h_ = 25.1 nm) in reassembly buffer C was comparable to that of wild‐type CCMV protein cages (*D*_h_ = 24.4 nm) in pH 4.9 buffer, and TEM images confirmed the successful and efficient encapsulation of the AuNPs (Figure S8c,d). To further assess the impact of encapsulated AuNPs on the dimensions of CCMV capsid, TEM images of mixed AuV and CCMV (1:1 molar equivalents) in reassembly buffer C were obtained (Figure S8e). Comparing the size distributions shows that AuV has a slightly smaller measured diameter (*D* = 24.8 nm) nm than swollen wild‐type CCMV (*D* = 26.4 nm), with no significant differences observed in surface morphology between the two (Figure S8f).

**Figure 3:**
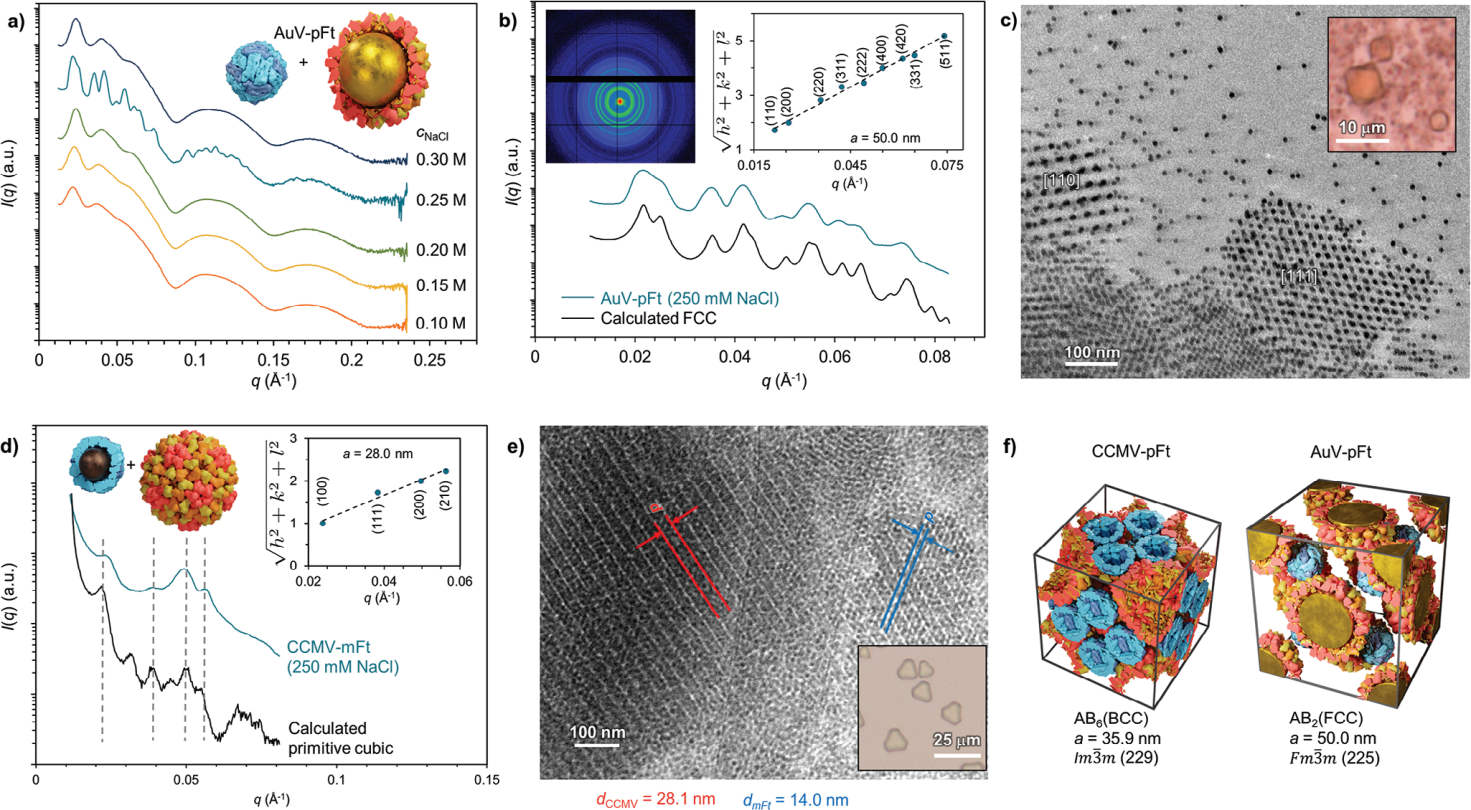
Structural characterization of AuV–pFt and CCMV‐mFt complexes. a) SAXS of AuV–pFt complexes measured at various ion strengths. b) SAXS data of AuV–pFt complexes at 250 mm NaCl, compared to the fitted FCC model (offset along the y‐axis for clarity). Inset: 2D scattering pattern (top left) and square root of the sum of the square of the Miller indexes of the assigned reflections for the FCC structure versus the measured q‐vector positions (top right), dashed line presents a linear fit, which yields a lattice parameter *a*_SAXS_ = 50.0 nm (for cubic structures *a *= 2π  √(h^2^ + k^2^ + l^2^)/*q*_(hkl)_). c) Cryo‐TEM image of AuV–pFt complexes at 250 mm NaCl. Inset: Optical microscopy image of AuV–pFt crystals showing octahedral habit and diameter approaching 10 µm. d) SAXS of CCMV‐mFt complexes measured at 250 mm NaCl, compared to the fitted primitive cubic (PC) model (offset along the y‐axis for clarity). Inset: Square root of the sum of the square of the Miller indexes of the assigned reflections for the PC structure versus the measured q‐vector positions. Dashed line presents a linear fit, which yields a lattice parameter *a*_SAXS_ = 28.0 nm. e) Cryo‐TEM image CCMV‐mFt complexes at 250 mm NaCl, the measured plane distances are outlined. Inset: Optical microscopy image of CCMV‐mFt crystals with sizes ≈10 µm. f) Comparison the unit cells of CCMV–pFt and AuV‐pFt crystals.

Next, we assembled the AuV cages with pFt directly in the reassembly buffer C (50 mm Tris‐HCl, 50 mm NaCl, 10 mm KCl, 5 mm MgCl_2_). The assembly conditions were optimized by testing various ionic strength conditions (**Figure** **3**a). The SAXS data for the AuV‐pFt binary crystal obtained at 250 mm NaCl shows a characteristic face‐centered cubic (FCC) crystal structure (space group *Fm3m*, number 225), with a lattice constant of *a* = 50.0 nm (Figure 3b). Given the significantly higher electron density of the gold nanoparticles compared to other constituents, this suggests that the AuV particles in this binary crystal adopt an FCC arrangement. The lattice constant of the AuV‐pFt FCC binary crystal is precisely √2times that of the CCMV‐pFt BCC binary crystal lattice constant. The change in the packing mode from BCC to FCC structure can be attributed to the different assembly conditions^[^ ^48^, ^49^, ^50^ ^]^ and the small change in the CCMV cage size and surface morphology caused by the AuNP encapsulation.^[^ ^51^, ^52^, ^53^ ^]^ Cryo‐TEM images also support the FCC crystal structure, aligning well with the SAXS data analysis (Figure S9a,b). An image of multiple small crystallites shows for example the expected FCC arrangement of AuV particles viewed along the [110], and [111] projection axes (Figure 3c; Figure S10).

However, the position of the pFt particles within the FCC structure of AuV‐pFt crystals is difficult to observe directly with SAXS or TEM. We therefore prepared pFt cages containing iron oxide nanoparticles (mFt) that are easier to detect. DLS measurements show that mFt has a similar size as the empty pFt cages (Figure S11a). Characterization by size‐exclusion chromatography and TEM also confirmed the successful encapsulation of iron oxide nanoparticles inside the Ft cage (Figure S11b,c).^[^ ^19^ ^]^ Binary crystal of CCMV‐mFt, which consists of empty CCMV capsid and mFt loaded with iron oxide particles was then prepared. SAXS analysis of samples prepared at 250 mm NaCl shows a primitive cubic lattice (space group *pm3m*, number 221), with a lattice constant of *a* = 28.0 nm (Figure 3d). Notably, the SAXS originates mostly from mFt rather than the native CCMV due to the markedly higher electron density of the iron oxide core compared to the RNA genome. The relative positioning and intensities of peaks assigned to (*hkl*) = (100), (111), (200), (210) match with a primitive cubic arrangement of mFt within the binary crystal. The crystal structure was further confirmed by Cryo‐TEM imaging, which shows 3D polycrystallites consisting of CCMV and mFt entities (Figure S12).

Remarkably, discernible CCMV planes were evident, each separated by ≈28.1 nm, whereas mFt planes had a spacing of 14.0 nm (Figure 3e). Moreover, a cryo‐TEM image, alongside its corresponding Fourier transform and inverse Fourier transform, provides a detailed view of the arrangement of CCMV‐mFt lattice (Figure S13a). The inverse Fourier transform image aligns well with the expected AB_2_^FCC^ model unit cell when viewed along the [111] projection axis (Figure S13b). This suggests that the CCMV within CCMV‐mFt crystals adopt an FCC arrangement. Likewise, the pFt particles within AuV‐pFt crystals may adopt a primitive cubic arrangement being therefore isostructural with the CCMV‐mFt crystals. Regardless of the type of nanoparticle incorporated into the cages the resulting heterogeneous binary crystals form a AB_2_^FCC^ lattice (Figure 3f). Taken together, these findings confirm that the binary protein cage systems loaded with single nanoparticle form an antifluorite structure similar to Mg_2_Si.^[^ ^54^ ^]^ Specifically, the negatively charged CCMV cages occupy the FCC lattice sites, while positively charged ferritin cages entities reside within the tetrahedral interstitial sites (Table S1). Additionally, hanging‐drop vapor diffusion was used to prepare large binary crystals 10 µm in diameter. The crystal shape resembles a halved octahedron sectioned in the [111] direction (Figure 3e Inset).

**Figure 4:**
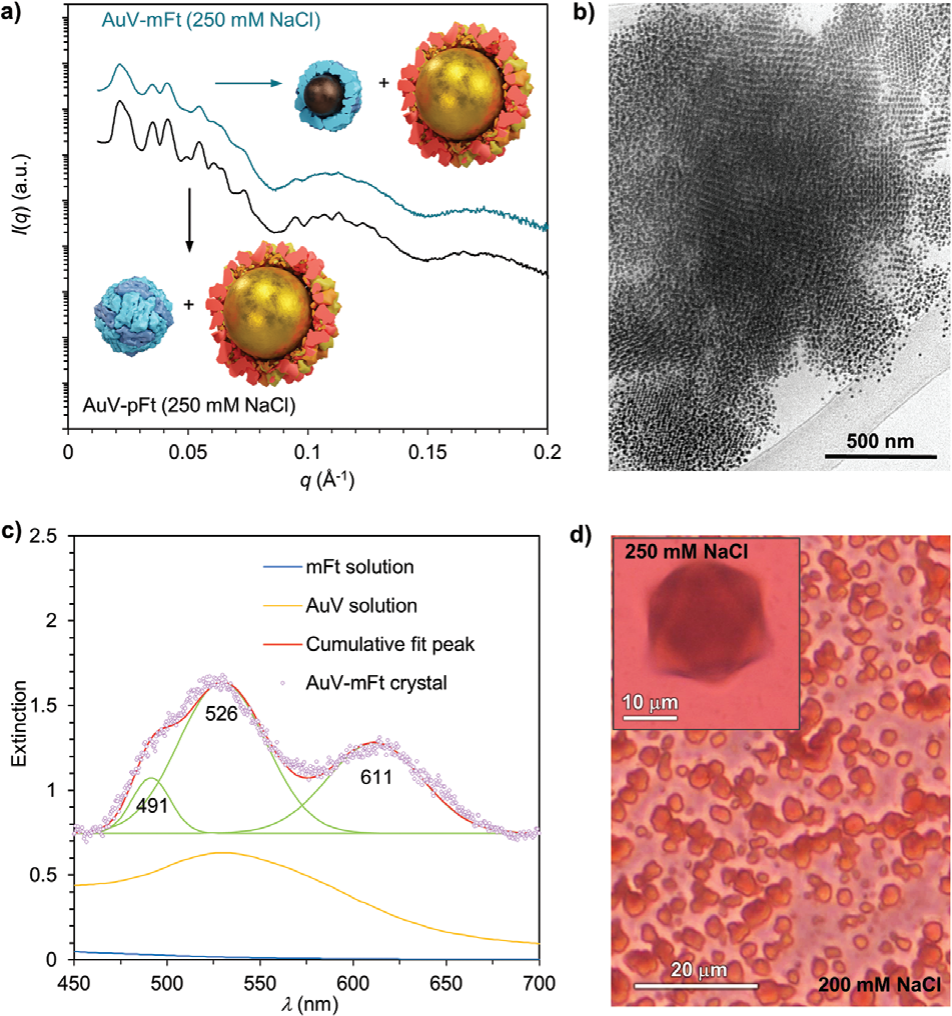
Structural characterization of AuV‐mFt complexes. a) The comparison of SAXS data between AuV‐pFt and AuV‐mFt complexes at 250 mm NaCl. b) Cryo‐TEM image of AuV‐mFt complexes at 250 mm NaCl. c) The optical spectrum of a single AuV‐mFt crystal, mFt, and AuV solution. d) Optical microscopy images of AuV‐mFt crystals.

Finally, we characterized the binary protein crystals containing both gold and iron oxide nanoparticles (AuV‐mFt). SAXS data shows that under 250 mm NaCl concentration, the integrated 1D curves of AuV‐mFt closely resembled those of AuV‐pFt (**Figure** **4**a). Cryo‐TEM images (Figure 4b; Figure S14) revealed single crystal domains exceeding 1 µm. Crystallites viewed along the [100] and [110] projection axes exhibited the anticipated FCC arrangement (Figure S15). This suggests that also the AuV‐mFt adopts an AB_2_^FCC^ antifluorite structure, similar to AuV‐pFt and CCMV‐mFt crystals. Given the distinctive optical properties arising from the surface plasmon resonance of gold nanoparticles, we employed absorption spectroscopy to gain further insights into the optical properties of the binary crystals. The analysis of transmitted light through an individual AuV‐mFt crystal revealed absorption spectra characterized by a triple peak. This spectral pattern can be modeled as a superposition of three Gaussian peaks centered at 491 , 526 , and 611 nm (Figure 4c). The major peak at 526 nm corresponds to the same wavelength observed for free AuV particles in solution, and the spectral feature of the other two peaks is consistent with theoretical predictions and previous demonstrations of plasmonic band splitting in metallo‐dielectric materials due to dipolar coupling among the gold cores.^[^ ^55^, ^56^ ^]^ Furthermore, AuV‐mFt crystals exhibited magnetic properties that are not present in free mFt particles. Crystals with diameters of ≈20 µm (Figure 4d Insert) and 3 µm (Figure 4d) can be prepared via hanging‐drop vapor diffusion at 250 mm NaCl and 200 mm NaCl separately. In aqueous solution, a mixture of free AuV and mFt particles at 300 mm NaCl cannot be attracted with a permanent magnet, however the crystals were easily attracted to the desired locations, as demonstrated in the supplemental video (Video S1).

## 3 Conclusion

In summary, we have demonstrated the design of binary superlattices guided by protein cages of distinct types. Several factors govern the formation of lattice structures, including particle charge, size, and surface morphology. Specifically, by optimizing the electrostatic assembly process, the empty cages (CCMV‐pFt) form an AB_6_^BCC^ type crystal structure. The internal cavities of CCMV and Ft provide an interesting platform for the formation of hybrid nanoparticle superlattices guided by the cages. We explored the assembly of 3D binary nanoparticle structures composed of 10 nm gold nanoparticles and 4 nm iron oxide nanoparticles. When protein cages encapsulating nanoparticles are employed for assembly, the resulting structure adopts an AB_2_^FCC^ configuration, which is isostructural with Mg_2_Si. Furthermore, we demonstrate that the protein cage‐directed assembly of the binary nanoparticle superlattice is more organized than our previous work on the assembly of positively charged AuNP and Ft,^[^ ^20^ ^]^ in the meanwhile, it shows a level of order comparable to the reported self‐assembly of oppositely charged inorganic nanoparticles.^[^ ^57^ ^]^ This improvement is attributed to the high homogeneity of the protein cages, allowing for the formation of crystals as large as several tens of micrometers. Hence, we anticipate that these heterogeneous binary crystals will not only advance our understanding of programmable multi‐component nanoparticle array fabrication, but also hold promise for future advancements in the field of magneto‐plasmonic materials.

## 5 Conflict of interest

The authors declare no conflict of interest.

6

## Supporting information



Supporting Information

Supplemental Video 1

## Data Availability

The data that support the findings of this study are available from the corresponding author upon reasonable request.
