# Supplementary material for: Protein Cage Directed Assembly of Binary Nanoparticle Superlattices
Source: Adv Sci (Weinh). 2024 Oct 14;11(45):2408416. doi: 10.1002/advs.202408416 (PMC11615748; doi:10.1002/advs.202408416)
Supplement: Supplementary file 1 — Supporting Information [file ADVS-11-2408416-s001.pdf]

ADVANCED SCIENCE
Open Access

Supporting Information

for *Adv. Sci.*, DOI 10.1002/advs.202408416

Protein Cage Directed Assembly of Binary Nanoparticle Superlattices

*Yu Zhou, Ahmed Shaukat, Jani Seitsonen, Carlo Rigoni, Jaakko V. I. Timonen and Mauri A. Kostiainen**

Materials and Methods:

Materials. CCMV was produced and isolated from California black-eye peas (Vigna unguiculata). 10 nm gold nanoparticles were supplied by Sigma Aldrich. Thiol modified DNA sample was purchased from Integrated DNA Technologies. Glass microscope slides and cover slips were purchased from VWR. Miscellaneous chemicals were purchased from Sigma Aldrich unless otherwise noted. MilliQ water with a resistivity > 18.2 MΩ·cm was used for all experiments.

Protein Expression and Purification. pFt backbone sequence was first optimized by OptimumGeneTM, thus synthesized and inserted into pET-25b(+) by GenScript company. *E. coli.* T7 Express strain (New England Biolabs) was chosen as the expression strain. For protein expression and purification, a similar method was adopted according to a previously published protocol. Eventually protein purity was analyzed by SDS-PAGE **(Fig. S1)**, and its concentration was determined with a NanoDrop LITE spectrophotometer (Thermo Scientific).

Synthesis of functionalized protein particles. Iron oxide loaded pFt (mFt): For the synthesis of pFt, a similar method was used according to a previously published protocol.[19] Concentration was estimated with the Bradford method and the final product was stored in 20 mM Tris-HCl (pH 7.4) at 4 °C until further usage.

10 nm Gold nanoparticles loaded CCMV (AuV): Approximately 0.1 ml of a 16 mg ml^{-1} stock of CCMV particles was dialyzed overnight at 4 °C against 1 L of disassembly buffer A (0.05 M Tris-HCl, pH 7.5, 0.5 M $CaCl_2$, 0.001 M dithiothreitol (DTT), and 0.0002 M phenylmethylsulfonyl fluoride (PMSF)) using a Slide-A-Lyser dialysis cassette (Thermo Fisher) with a 3.5 kDa molecular weight cut-off. The RNA–protein mixture obtained was centrifuged at 4 °C at 21000×g for 6 h and the upper 3/4 of the supernatant, containing the purified protein, was then collected, while the lower 1/4 volume including the yellowish RNA pellet located at the bottom of the centrifuge tube was discarded. The collected supernatant was then dialyzed against 1 L of buffer B (0.02°M Tris-HCl, pH 7.5, 1 M NaCl, 0.001 M DTT, and 0.0002 M PMSF) for 12 h at 4 °C. The ratio Abs280/Abs260 was used to determine the amount of nucleic acid contamination, and only the sample with a value above 1.5 can be used for the next step. Purified, disassembled capsid protein was stored at 4 °C in buffer B for further encapsulation of gold nanoparticles (AuNPs).

To enhance the loading efficiency of AuNPs into CCMV cages, a modification of single-strand DNA (ssDNA) on the surfaces of AuNPs was employed. A published pH-assisted and surfactant-free route was adapted with slight modifications for the purpose.[44] Briefly, thiol-modified ssDNA in MilliQ water was treated with tris(2-carboxyethyl)phosphine (TCEP) (100×) for 1 h at room temperature before usage. This step effectively cleaved the existing disulfide bonds. Subsequently, 2.5 μL of treated thiol-modified ssDNA (200 μM) was added into 0.5 mL of 10 nm AuNPs (10 nM, stabilized suspension in citrate buffer). Next, a small volume (50 μL) of 500 mM citrate-HCl buffer (pH 3) was rapidly added to the DNA/AuNP mixture followed by pipette mixing. After incubation at room temperature for 1 h,

the prepared AuNP-ssDNA conjugates were harvested through centrifugation (15000 rpm, 30 min, 4 °C), and the precipitate was rinsed three times with MilliQ water and stored at 4 °C. The successful attachment of ssDNA was confirmed by both DLS and TEM characterization.

For the encapsulation of ssDNA modified AuNPs into CCMV during the reassembly of protein cages, the capsid protein concentration was adjusted to 0.50 mg/mL using buffer B without DTT and PMSF. In the meanwhile, the ssDNA modified AuNPs were measured, evaporated and redissolved into 50 µL buffer B without DTT and PMSF. Next, AuV were assembled using capsid proteins and AuNPs in a molar ratio of 270:1 in a total volume of 300 µL. This mixture was dialyzed against reassembly buffer C (50 mM Tris-HCl, 50 mM NaCl, 10 mM KCl, 5 mM $MgCl_2$) for 12 h at 4 °C. Afterward, the product was concentrated to the desired concentration using an Amicon ultra centrifugal filter (Millipore) with a 100 kDa molecular weight cut-off. The size of the synthesized AuV and the efficacy of encapsulation were confirmed by TEM characterization.

Dynamic light scattering (DLS). A DLS device (Zetasizer Nano ZS Series, Malvern Instruments) equipped with a 4 mW He–Ne ion laser at a wavelength of 633 nm and an Avalanche photodiode detector at an angle of 173° was used to measure the hydrodynamic diameter (D_h) of assemblies. Experiments were carried out at 25 °C and PMMA cuvettes were used for all of the measurements. For titration series, 0.1 mg mL^{-1} of CCMV dissolved in buffer (20 mM sodium acetate, pH 4.8) was titrated with the pFt (0.1–16 mg mL^{-1} in 20 mM Tris-HCl, pH 7.4) to reach the desired ratio (no dilution correction was done as the total addition did not exceed 5% of sample volume), which was finally titrated with 0.01–0.2 M NaCl to disassemble the complex. Zetasizer software (Malvern Instruments) was used to obtain the scattering intensity (count rate), and particle size distributions.

Small-angle X-ray scattering (SAXS). The Xenocs Xeuss 3.0 C device, equipped with a GeniX 3D Cu microfocus source (with a wavelength of $\lambda = 1.542$ Å), and an EIGER2 R 1M hybrid pixel detector, was used to measure the SAXS samples. The measurements were conducted at a sample-to-detector distance of 1.1 m. To obtain one-dimensional SAXS data, the 2D scattering data was azimuthally averaged. The magnitude of the scattering vector q can be calculated using the equation $q = 4\pi \sin\theta / \lambda$, where 2θ represents the scattering angle. The theoretical SAXS patterns were computed using XSACT software. This involved utilizing signals from both free particles as form factors, alongside the cell parameter and FWHM of Bragg peaks to optimize the curves.

For sample preparation, 3 µL NaCl water solution was added into 1 µL pFt solution (4 mg mL^{-1} in 20 mM Tris-HCl, pH 7.4) to adjust the ionic strength after which 4 µL CCMV solution (4 mg mL^{-1} in buffer C) was added under stirring.

Electron microscopy imaging. Conventional transmission electron microscopy (TEM) imaging of individual protein cages was carried out with Tecnai 12 Bio-Twin transmission electron microscope (FEI) using an acceleration voltage of 120 kV. The sample was added on Formvar/Carbon Supported Copper Grids and the excess sample was blotted away with filter paper. The grids were then washed

with milliQ water, followed by 1 wash and 1 incubation (30 s) on 2% uranyl acetate drops. Uranyl acetate incubations were omitted for unstained samples.

The Cryogenic transmission electron microscopy (Cryo-TEM) images were obtained using a JEM 3200FSC field emission microscope (JEOL) operating at 300 kV in bright field mode, equipped with an Omega-type zero-loss energy filter. Gatan Digital Micrograph software was utilized to capture the images while maintaining the specimen temperature at -187 °C. To prepare the Cryo-TEM samples, a 3 μL aqueous dispersion of the sample was placed on a 200-mesh Lacey carbon film mounted on Copper TEM Grids (agar scientific). These grids were then rapidly frozen by plunging them into liquid ethane using a Leica grid plunger. The blotting process lasted for 3 seconds under 100% humidity. Subsequently, the grids containing the vitrified sample solution were kept at liquid nitrogen temperature and transferred to the microscope under cryogenic conditions. Prior to usage, all the TEM grids were subjected to plasma cleaning (NanoClean 1070, Fischione Instruments). ImageJ software was employed for further processing of the acquired images.

Cryo Electron Tomography (Cryo-ET). Single-axis tilt series images at 2° increments were acquired in low-dose mode with serialEM software. The total electron dose during the recording phase was ~40 e/Å^2. The tilt series images were aligned and 3D reconstructed with IMOD/Etomo program suite. 3D tomographic reconstructions were finally visualized and rendered with Chimera.

Optical microscopy. Samples for optical microscopy were readied through the hanging drop technique. A cover slip (18 x 18 mm, VWR) was utilized as a platform to support the droplet above the reservoir media. This droplet held the same components as employed for SAXS measurements. The reservoir media was composed of 300 µL of 20 mM Tris buffer at pH 7.5. To prevent solvent evaporation, the cover slip bearing the sample was affixed to the reservoir cap using high vacuum glue, with the sample droplet oriented towards the sealed side. Afterward, the samples were left to incubate at room temperature for a duration of three days prior to imaging. The Zeiss Axio Vert A1 inverted microscope was utilized for optical microscopy imaging. The samples were imaged directly on the glass slides used during the crystallization process, without any additional preparation.

Single crystal Spectroscopy. Transmission spectra were obtained from single crystals submerged in the crystallization liquor using an inverted Zeiss Imager.Z1 microscope equipped with a Thorlabs MNWHL4 light source and an Andor Shamrock SR-193i-B1 spectrometer. The microspectroscopy was conducted using one microscope objective: Zeiss LD A-Plan 40x/0.50 Ph2. The width slit of the spectrometer was set to 20 μm, and the spectrum was calibrated using a multi bandpass filter (89101m Chroma). Spectral images were captured using an Andor Zyla sCMOS camera. Each spectral image contained the spectrum values in the y-axis corresponding to each position along the length of the slit in the x-axis. Once collected, the sample spectral images were opened with ImageJ and each spectrum intensity profile of was obtained by averaging the values of 20 pixels of the spectral images along the x-axis (this was well within the width of the single crystal in the case of the sample). The data was then

transferred to OriginPro for plotting and further elaboration. The value of absorbance was obtained by comparing the spectrum of the single crystal with the spectrum of the surrounding crystallization liquor.

Protein Sequences. pFt sequence used in this study was from FtnQC12.[36] The nine mutated residues have been marked in red.

MTTASTSQVRQNYHQDSEKAINRQIRLELYASYVYLSMSYYFDRDDVALKNFAKYF
LHQSHEEREHAEKLMKLQNQRGGRIFLQDIQKPDKDDWESGLRAMEKALKLEKKV
NQSLLELHKLATKKNDPHLCDFIETHYLNEQVKAIKELGDHVTNLRKMGAPRSGLA
EYLFDKHTLGDSDNES

Supplementary Figures:

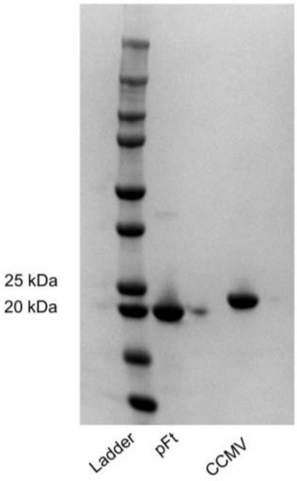

Figure S1. Coomassie-stained 12% SDS-PAGE characterization of pFt and CCMV used in this study.

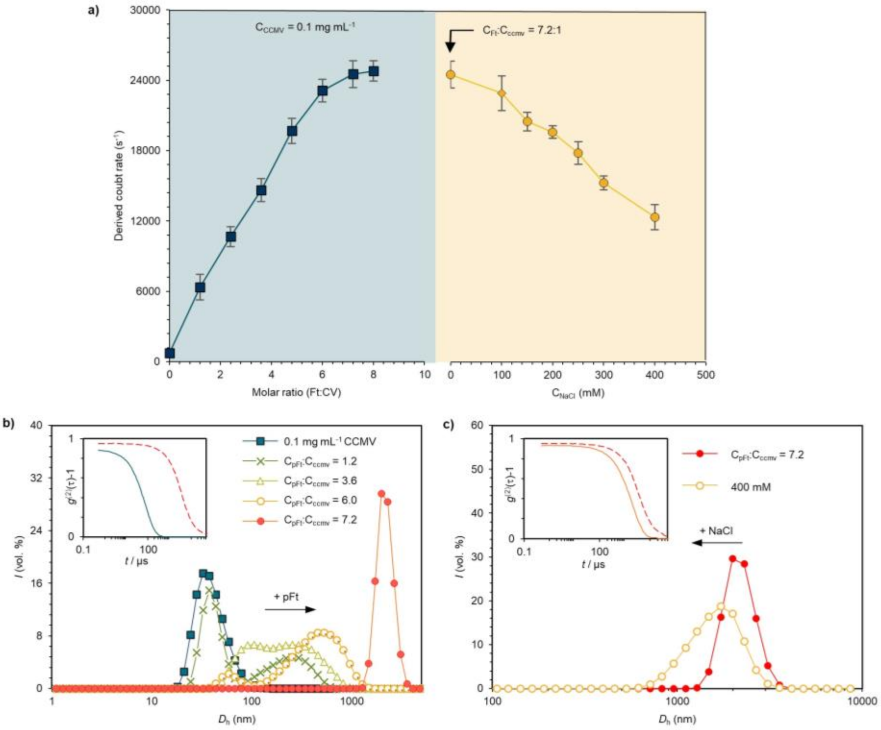

Figure S2. DLS characterization of the CCMV-pFt assembly and disassembly at pH 7.4. (a) Particles count rate data of the assembly and attempted disassembly process ate pH 7.4. (b-c) Volume-weighted size distribution of free CCMV titrated with increasing pFt, the resulting complexes cannot be disassembled with NaCl as in pH 7.4 buffer. insets: second-order autocorrelation functions of the corresponding measurements.

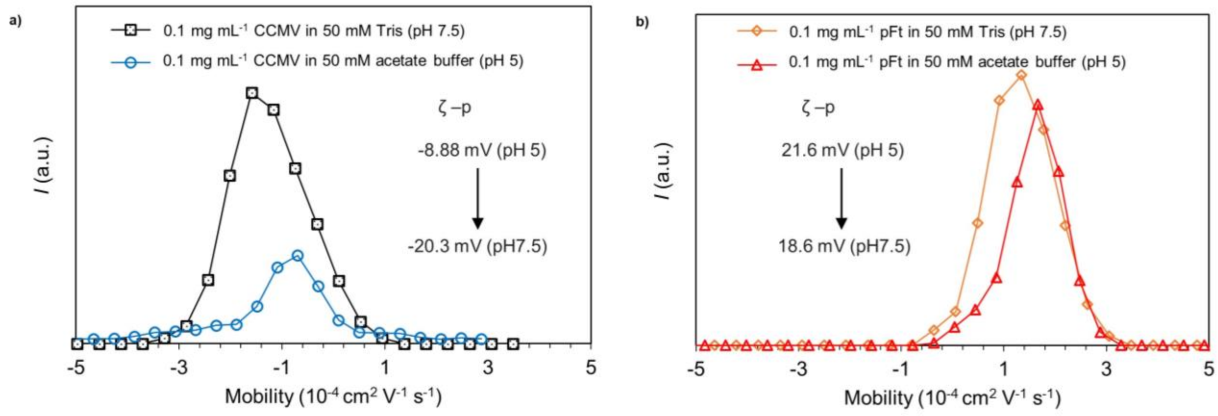

Figure S3. Electrophoretic mobility and ζ-p measurements for (a) CCMV and (b) pFt cages at different pH.

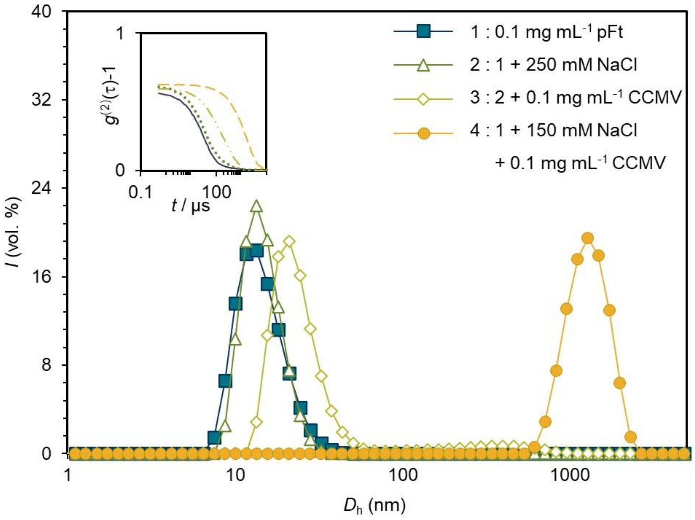

Figure S4. DLS characterization of the CCMV-pFt assembly at pH 7.4 with a different sequence of adding materials. pFt was first dispersed in the pH 7.4 buffer with the desired concentration of NaCl, thus the CCMV was introduced into the system. In this manner, 250 mM NaCl is sufficient to separate the protein particles by blocking the charge.

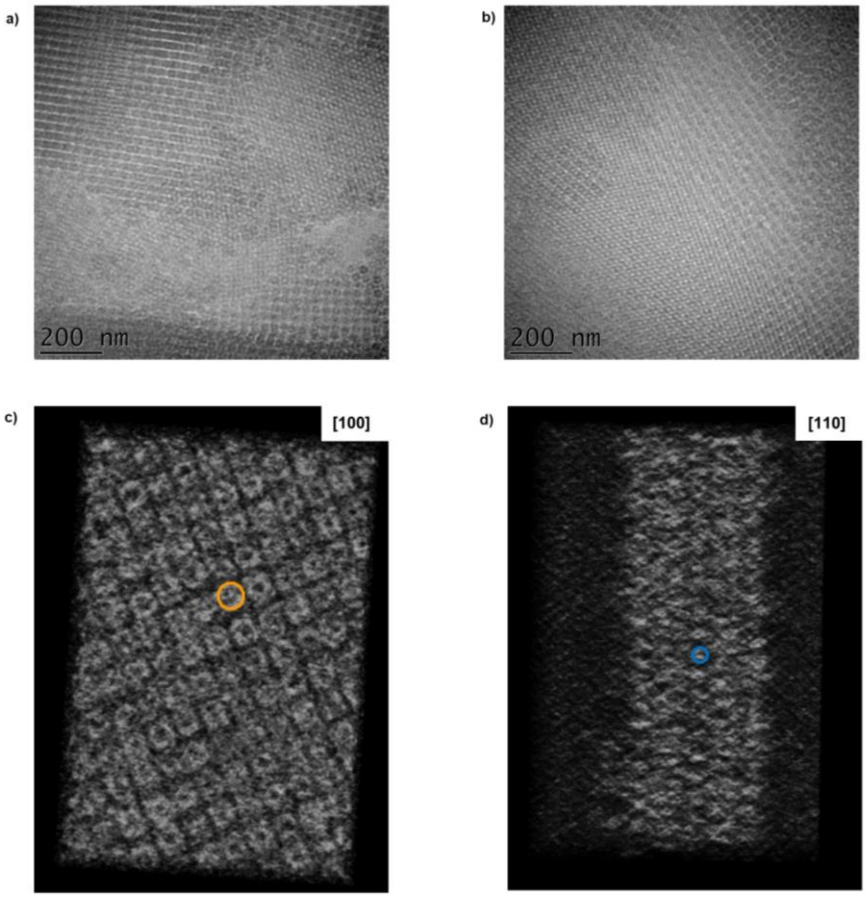

Figure S5. Cyro-TEM characterization of CCMV-pFt complexes at 200 mM NaCl. (a-b) Cryo-TEM images of the vitrified aqueous solution containing complex which present CCMV and pFt layers at the same time, scale bars are 200 nm. (c-d) Electron density maps obtained by Cryo-ET, which are viewed along the [100], and [110] zone axis respectively, CCMV (orange) and pFt (blue) particles are labeled to demonstrate differences in particle size.

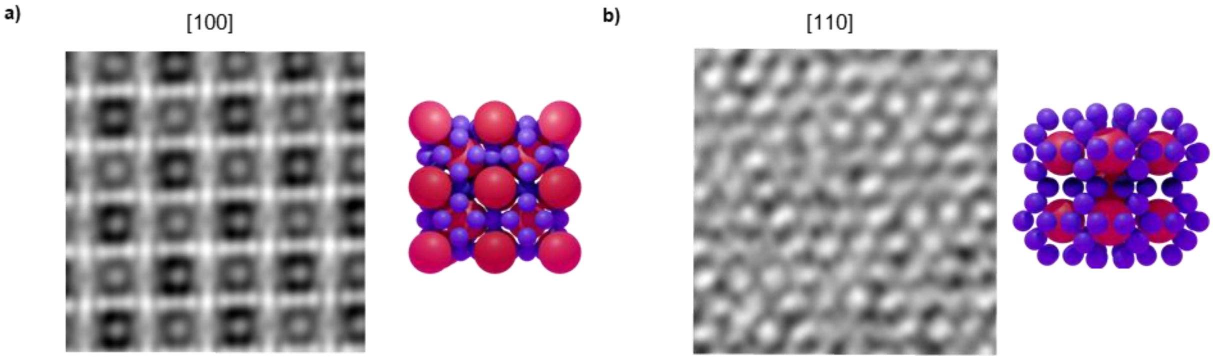

Figure S6. Inverse Fourier transform calculated with selected Fourier components of (a) CCMV layer lattice form Figure 2(d), (b) pFt layer lattice form Figure 2(e) separately. The models of unit cells viewed along the [100] and [110] projection axe are presented as comparison. Images are 150 nm x 150 nm.

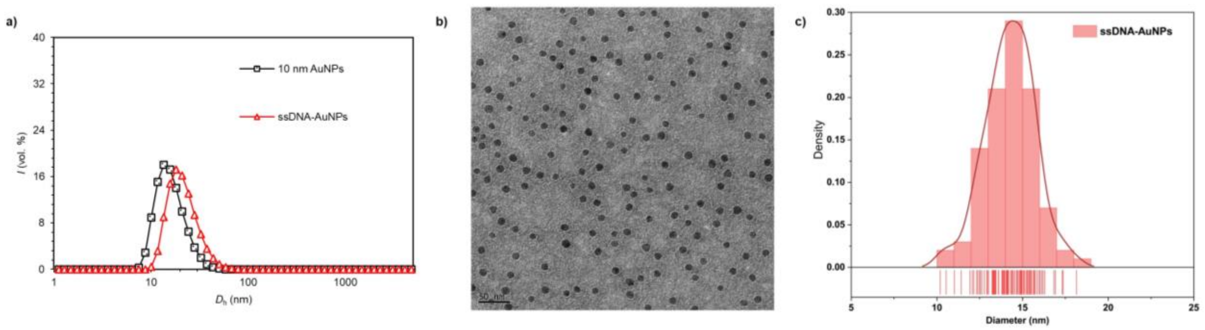

Figure S7. Characterization of the gold nanoparticles attached with the ssDNA. (a) Volume-weighted hydrodynamic diameters of 10 nm AuNPs and ssDNA-AuNPs determined by DLS. (b) TEM analysis of synthesized ssDNA-AuNPs, scale bar is 50 nm. (c) Observed size distributions (in diameter) from b) for ssDNA-AuNPs (red) particles.

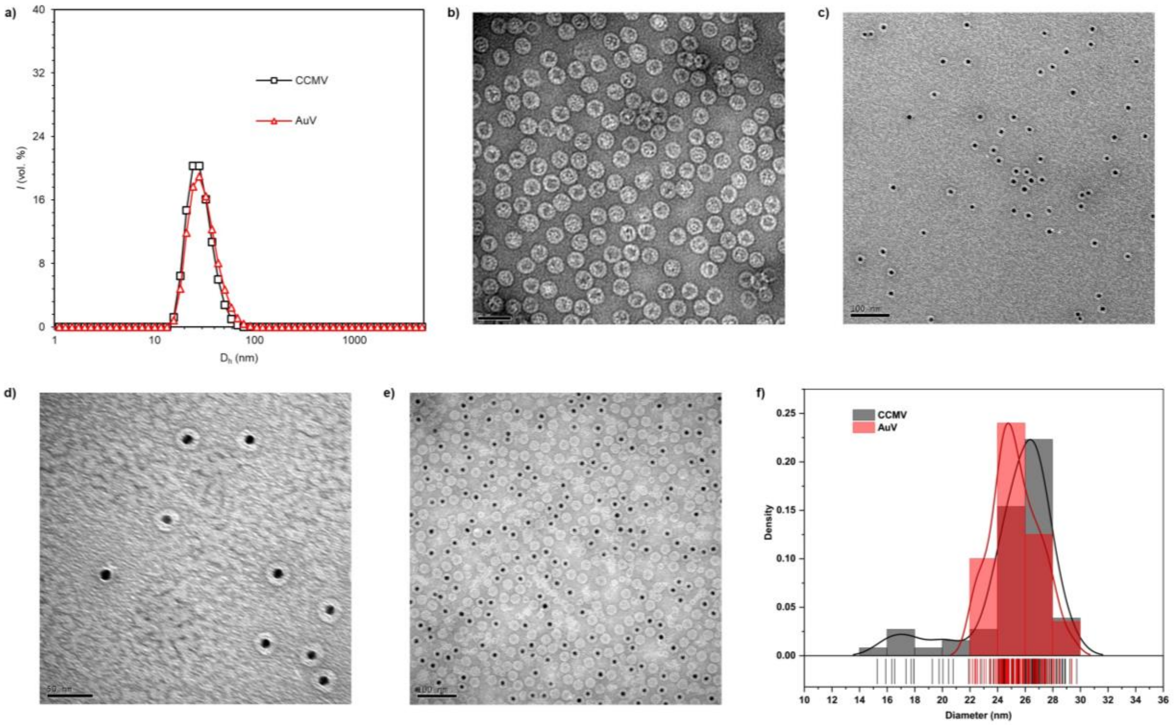

Figure S8. Characterization of the CCMV cages containing gold nanoparticles. (a) Volume-averaged hydrodynamic diameters of CCMV and AuV particles determined by DLS. (b) TEM analysis of native CCMV in pH 4.9 buffer, scale bar is 50 nm. (c-d) TEM analysis of AuV particles in reassembly buffer C, scale bars are 100 nm and 50 nm. (e) TEM analysis of a mixture of native CCMV and AuV particles in reassembly buffer C, scale bar is 100 nm. (f) Observed size distributions (in diameter) from e) for native CCMV (black) and AuV (red) particles.

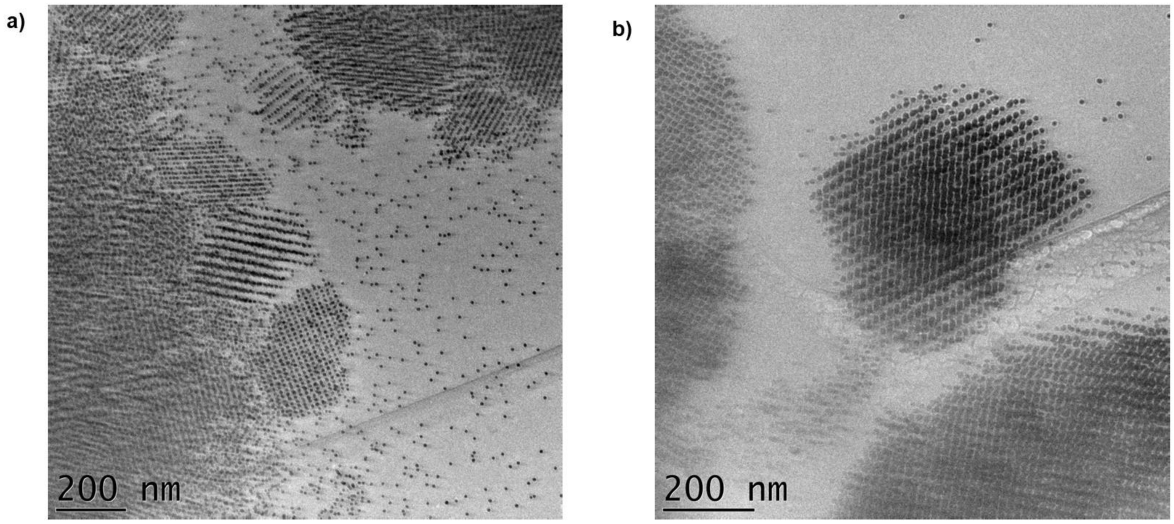

Figure S9. (a-b) Additional Cyro-TEM characterizations of the AuV–pFt complexes, scale bars are 200 nm.

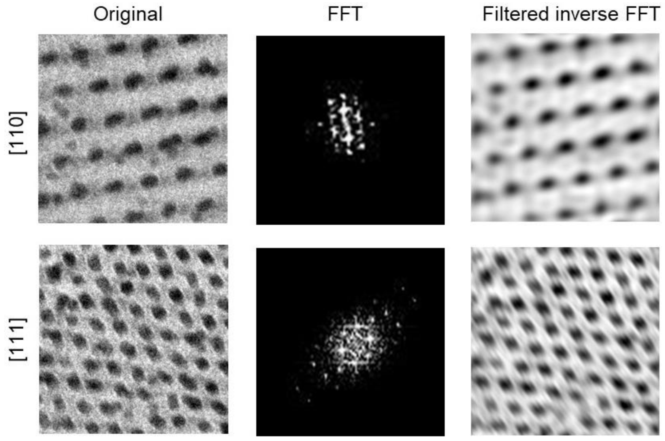

Figure S10. TEM characterization of the AuV-pFt superlattice structures. Each panel showcases a Cryo-TEM image, alongside its corresponding image Fourier transform and inverse Fourier transform calculated with selected Fourier components. All images are depicted at a consistent scale of 150 nm × 150 nm.

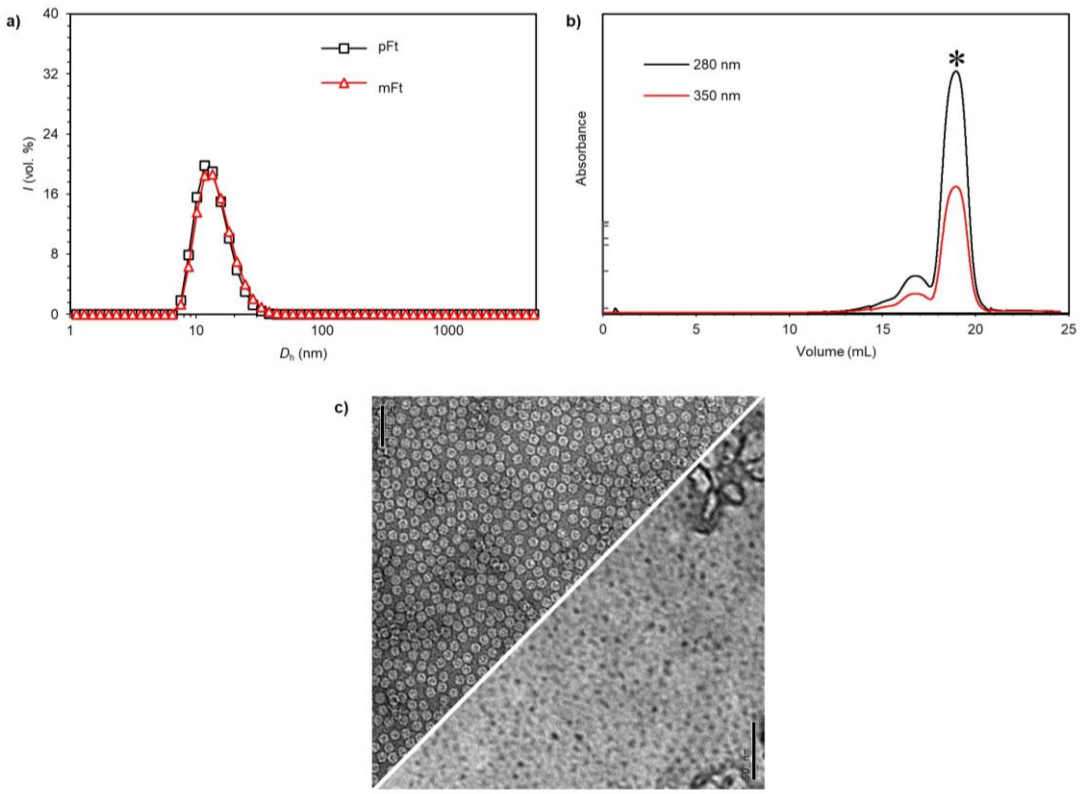

Figure S11. Characterization of the mFt cages containing iron oxide nanoparticles. a) Intensity-weighted hydrodynamic diameters of pFt and mFt particles determined by DLS. b) Size-exclusion chromatogram of mFt after iron oxide nanoparticle synthesis, which monomeric protein container fraction is marked with asterisk (*). c) TEM analysis of mFt nanoparticles, upper left panel shows micrograph with negative staining agent applied, lower right panel is without staining agent. Scale bars are 50 nm.

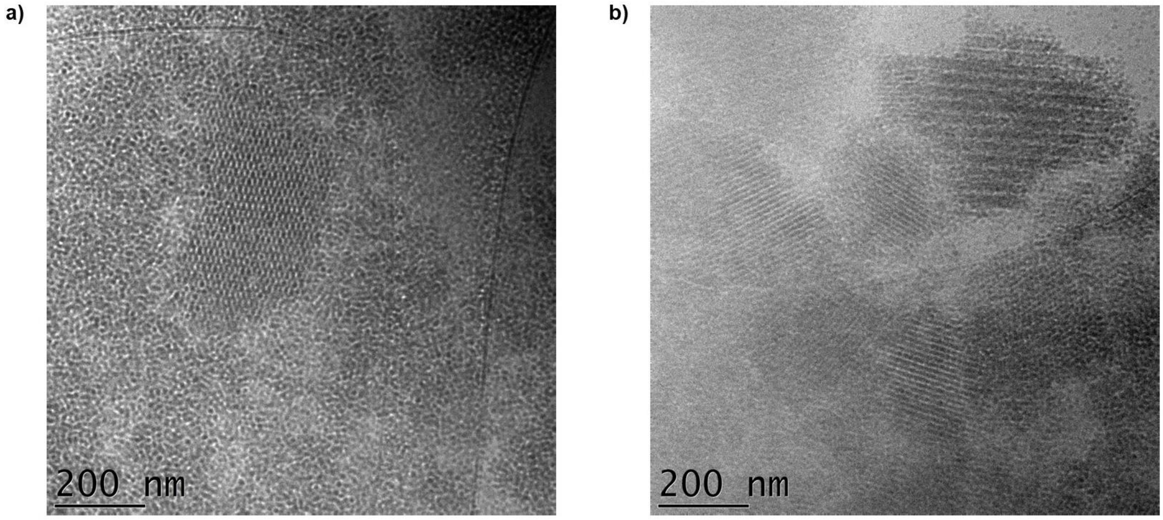

Figure S12. (a-b) Additional Cyro-TEM images of the CCMV–mFt complexes, scale bars are 200 nm.

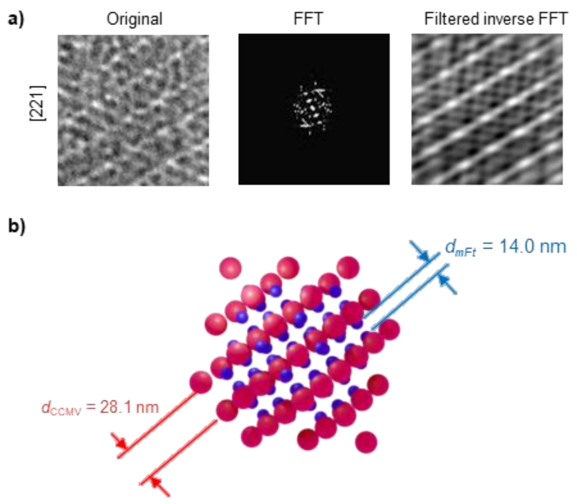

Figure S13. TEM characterization of the CCMV-mFt superlattice structures. (a) Each panel showcases a Cryo-TEM image, alongside its corresponding image Fourier transform and inverse Fourier transform calculated with selected Fourier components. All images are depicted at a consistent scale of 150 nm × 150 nm. (b) A crystal model viewed along [221] projection axis.

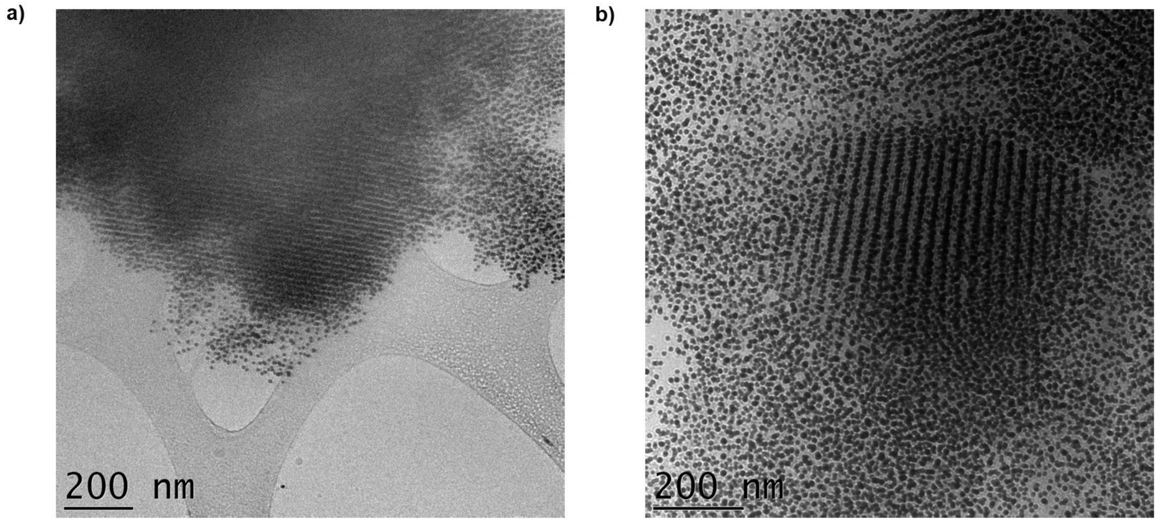

Figure S14. (a-b) Additional Cyro-TEM images of the AuV-mFt complexes, scale bars are 200 nm.

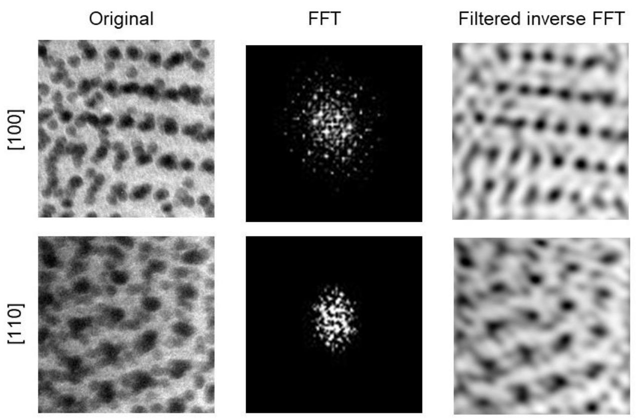

Figure S15. TEM characterization of the AuV-mFt superlattice structures. Each panel showcases a Cryo-TEM image, alongside its corresponding image Fourier transform and inverse Fourier transform calculated with selected Fourier components. All images are depicted at a consistent scale of 150 nm × 150 nm.

2. Supplementary Table:

	$(CCMV\text{-}pFt_6)^{bcc}$	$(AuV\text{-}Ft_2)^{fcc}$
**Bravais lattice**	*body-centered cubic*	*face-centered cubic*
**Space group (number)**	$Im\bar{3}m$ (229)	$Fm\bar{3}m$ (225)
**Unit cell size (lattice parameter *a*)**	35.9 nm	50.0 nm
**Protein cage centre-to-centre distance**	CCMV to CCMV: 31.1 nm<br>pFt to pFt: 12.7 nm<br>CCMV to pFt: 20.1 nm	AuV to AuV: 35.4 nm<br>Ft to Ft: 28.0 nm<br>AuV to Ft: 21.7 nm
**Particles in unit cell**	14 (2 CCMV, 12 pFt)	12 (4 AuV, 8 Ft)
**Primitive vectors x,y,z**	$\mathbf{A_1} = -\frac{1}{2}a\mathbf{X} + \frac{1}{2}a\mathbf{Y} + \frac{1}{2}a\mathbf{Z}$<br>$\mathbf{A_2} = \frac{1}{2}a\mathbf{X} - \frac{1}{2}a\mathbf{Y} + \frac{1}{2}a\mathbf{Z}$<br>$\mathbf{A_3} = \frac{1}{2}a\mathbf{X} + \frac{1}{2}a\mathbf{Y} - \frac{1}{2}a\mathbf{Z}$	$\mathbf{A_1} = \frac{1}{2}a\mathbf{Y} + \frac{1}{2}a\mathbf{Z}$<br>$\mathbf{A_2} = \frac{1}{2}a\mathbf{X} + \frac{1}{2}a\mathbf{Z}$<br>$\mathbf{A_3} = \frac{1}{2}a\mathbf{X} + \frac{1}{2}a\mathbf{Y}$
**Basis vectors x,y,z (anionic species) (cationic species)**	$\mathbf{B_1} = (0\mathbf{X}, 0\mathbf{Y}, 0\mathbf{Z})$<br>$\mathbf{B_2} = (0\mathbf{X}, 0.25\mathbf{Y}, 0.5\mathbf{Z})$	$\mathbf{B_1} = (0\mathbf{X}, 0\mathbf{Y}, 0\mathbf{Z})$<br>$\mathbf{B_2} = (0.25\mathbf{X}, 0.25\mathbf{Y}, 0.25\mathbf{Z})$
**Unit cell image CCMV: orange Cationic ferritin : blue**	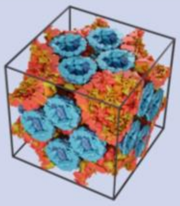	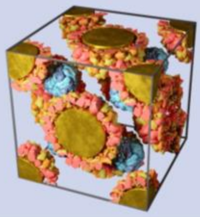

Table S1. Unit cell details for the suggested binary structures.
